# Acute Peripheral Facial Palsy after Chickenpox: A Rare Association

**DOI:** 10.1155/2014/754390

**Published:** 2014-06-25

**Authors:** Helena Ferreira, Ângela Dias, Andreia Lopes

**Affiliations:** Departamento de Pediatria, Centro Hospitalar do Alto Ave, Rua dos Cutileiros, Creixomil, 4835-044 Guimarães, Portugal

## Abstract

Chickenpox, resulting from primary infection by the varicella-zoster virus, is an exanthematous disease very common during childhood and with good prognosis. However, serious complications, namely, neurological syndromes, may develop during its course, especially in risk groups, including adolescents. Peripheral facial palsy is a rare neurologic complication that has been previously described. *Conclusion*. We report the case of a teenager with peripheral facial palsy as a complication of chickenpox, aiming to increase the awareness of this rare association.

## 1. Introduction

Varicella-zoster virus (VZV) is a human herpesvirus which leads to the onset of two distinct diseases: varicella (or chickenpox) and herpes zoster (or shingles). Primary VZV infection results in chickenpox, which normally manifests itself as a generalized exanthematous rash. The virus then becomes latent in dorsal root ganglia and can manifest itself later in life as shingles, which is characterized by a painful rash with blisters limited to one or more adjacent sensory dermatomes [1–4]. Generally, chickenpox is a benign and self-limited infection with good prognosis; however, severe complications may arise [3, 5]. The most common complication is bacterial superinfection of the skin, lungs, or bones [3, 6]. Neurological complications develop in up to 0.03% of the cases [4, 7, 8]. The main neurological syndromes are encephalitis, acute cerebellar ataxia, myelitis, and meningitis [2]. Peripheral facial palsy (PFP) is a rare neurologic complication of chickenpox, which may develop five days before to sixteen days after appearance of exanthema [7, 9].

Information about PFP as a complication of chickenpox is scarce and there are no guidelines about its optimal clinical management.

Here, we report a case of PFP following chickenpox and discuss the association between these clinical entities.

## 2. Case Report

A 15-year-old girl was admitted into the pediatric emergency department with drooping of the left corner of the mouth and inability to close the right eyelid for 3 days.

Her personal and family history were unremarkable.

Two weeks before she had developed a dental pain and underwent antibiotic treatment for 3 days, suspended by self-initiative due to the appearance of fever and an exanthematous rash compatible with chickenpox, she did not undergo acyclovir treatment.

Furthermore, there was no clinical history of associated headache, nausea, vomiting, visual disturbances, neurological focal deficits, muscular strength, sensibility asymmetries, or other symptoms suggestive of an additional central nervous system involvement. Retroauricular pain, hyperacusis, decreased production of tears, or altered taste was also denied. There was no history of previous cold exposure.

The patient was afebrile and her vital signs were within the normal range, namely, her blood pressure which was 110/75 mmHg (below the 90th percentile for gender, age, and height). There were multiple crusted lesions scattered all over the body. She had no vesicular eruption over the external pinna, ear canal, or pharynx. Otoscopy revealed a gray and translucent tympanic membrane in the neutral position. There were no signs of otitis or mastoiditis or evidence of ear trauma. No evidence of dental abscess was found. The ophthalmological examination revealed symmetrical pupillary reflexes, normal eye movements, and preserved convergence. The neurological examination showed asymmetries in the ability to perform eyelid closure and asymmetries in labial movements, which were not evident at rest due to a normal facial symmetry. The observed decreased ability to close the right eyelid and concomitant drooping of the left corner of the mouth were compatible with a right PFP as a neurological complication of chickenpox.

As chickenpox and PFP are two clinical diagnoses and this adolescent had pathognomonic signs and symptoms of chickenpox complicated by PFP, no other serologic or imaging studies were performed.

She was treated with acyclovir (20 mg/kg/dose, 4 times/day, during 5 days), artificial tears, and physical rehabilitation. During follow-up, a complete recovery of the initial deficits and a restoration of normal functions were registered.

## 3. Discussion

Chickenpox is a highly infectious acute, febrile, and exanthematous disease, affecting a great percentage of people during their lifetime, with greater incidence during childhood [3]. Because of its characteristic rash and distribution, along with epidemiologic information, the diagnosis is mainly clinical. Laboratory diagnosis is facilitated by the accessibility of the virus in superficial skin lesions but it is only justifiable if clinical doubt exists [3].

Chickenpox has generally a benign course although it can be associated with several complications, depending on immune status, presence of chronic diseases, and age [5]. Neurological complications associated with chickenpox are rather uncommon, being estimated in 1–3 per 10.000 cases [4]. The expected proportion of neurological complications among hospitalized children varies between 13.9% and 20.4% [5]. Encephalitis, acute cerebellar ataxia, myelitis, and meningitis are the most common neurological complications reported [2]. More uncommon are Guillain-Barré syndrome, meningoencephalitis, ventriculitis, optic neuritis, delayed contralateral hemiparesis, peripheral motor neuropathy, cerebral angiitis, Reye's syndrome, and facial palsy [4, 8].

The prevalence of neurological complications of chickenpox is highly variable, and few studies have been published in the pediatric population. A study with sixty cases of children and adults with neurological complications due to chickenpox reported encephalitis in 23.3% of those patients, cerebellar ataxia in 21.7%, meningitis in 8.3%, stroke in 13.3%, and PFP in 8.3% [10]. In a more recent study with pediatric patients only, the main neurological complication was cerebritis in 44.7%, followed by seizures in 22.3% and by meningoencephalitis, meningitis, and encephalitis in 10.5%. PFP was diagnosed in just 5.2% of children [5].

PFP may occur before, during, or after exanthema appearance. This peripheral neuropathy can be isolated or bilateral and can have different degrees of functional impairment [7–9, 11].

The relationship between PFP and chickenpox is neither common nor completely understood. Two possible mechanisms exist: direct nerve lesion due to direct viral toxicity or nerve damage associated with immunologically mediated inflammatory response [11].

Established guidelines for the treatment of varicella-zoster related neurological complications do not exist and therefore the treatment must be individualized for each patient. In the majority of the published reports, the pediatric patients were treated with acyclovir and/or steroids [7–9, 11].

In the present case, given the minimal neurological compromise, the treatment was carried out using oral acyclovir without steroids and artificial tears along with physical rehabilitation. After 5 days of treatment the adolescent had significantly recovered from her previous deficits, which supports the efficacy of the applied therapeutic measures.

Although scarce, the available reports on the prognosis of PFP associated with chickenpox show a good prognosis and 80% of the afflicted patients recover completely even without treatment. Yet, acyclovir and/or steroids might accelerate the expected recovery [8, 9].

Some investigations have been developed to determine the epidemiologic effect of varicella vaccine introduction in vaccination programs. These studies confirm that after the inclusion of this vaccine in the childhood vaccination schedule, the incidence of this disease has diminished drastically, not only in vaccinated individuals but also in the unvaccinated ones, due to herd immunity. The authors have also shown that the rates of hospitalization markedly declined as well as the complicated forms of chickenpox [12, 13]. A countrywide sentinel surveillance system was initiated in Germany after implementation of routine varicella vaccination. In that survey the number of reported varicella cases as well as chickenpox complications decreased by 63% and 81%, respectively, and neurologic complications accounted for only 0.03% of all cases [14].

In Portugal, this vaccine is not included in the national vaccination program. Consequently, the vaccination rate is very low, not being enough to generate herd immunity. Thus, prevalence remains high, and complications continue to emerge. In an attempt to reduce varicella complications, it is essential to vaccinate high-risk groups and to initiate antiviral drugs to those who develop the disease.

In conclusion, VZV can be a causative agent of PFP in the pediatric population. Despite being rare, this neurological complication of chickenpox should be kept in mind. The antiviral treatment should be highlighted as a fundamental therapeutic measure which can reduce the duration of symptoms and avoid possible complications following infection by the VZV.

## Abbreviations

Kg: Kilogram
mmHg: Millimeters of mercury
mg: Milligram
PFP: Peripheral facial palsy
VZV: Varicella-zoster virus.

## References

[1] Albrecht MA (2013). Clinical features of varicella -zoster virus infection: chickenpox. http://www.uptodate.com/online.

[2] Amlie-Lefond C, Jubelt B (2009). Neurologic manifestations of varicella zoster virus infections. *Current Neurology and Neuroscience Reports*.

[3] Gershon AA (2008). Varicella-zoster virus infections. *Pediatrics in Review*.

[4] Gnann JW (2002). Varicella-zoster virus: a typical presentations and unusual complications. *Journal of Infectious Diseases*.

[5] Bozzola E, Tozzi AE, Bozzola M (2012). Neurological complications of varicella in childhood: case series and a systematic review of the literature. *Vaccine*.

[6] Riaza Gómez M, de la Torre Espi M, Mencia Bartolome S, Molina Cabanero JC, Tamariz-Martel Moreno A (1999). Complications of varicella in children. *Anales Españoles de Pediatría*.

[7] Yilmaz C, Çaksen H (2005). Severe neurological complications of chickenpox: report of four cases. *European Journal of General Medicine*.

[8] Rama Rao G, Amareswar A, Kishan Kumar Y, Rani R (2008). Isolated facial palsy in varicella. *Indian Journal of Dermatology, Venereology and Leprology*.

[9] Ödemis E, Türkay S, Tunca A, Karadag A (2004). Acute peripheral facial palsy during chickenpox in a child. *Journal of Pediatric Neurology*.

[10] Shiihara H (1993). Neurological complications of varicella-zoster virus (VZV) infection. *No To Hattatsu*.

[11] Muñoz-Sellart M, García-Vidal C, Martnez-Yelamos S, Niub J, Fernndez-Viladrich P (2010). Peripheral facial palsy after varicella. Report of two cases and review of the literature. *Enfermedades Infecciosas y Microbiologia Clinica*.

[12] García Cenoz M, Castilla J, Chamorro J (2013). Impact of universal two-dose vaccination on varicella epidemiology in Navarre, Spain, 2006 to 2012. *Eurosurveillance*.

[13] Siedler A, Arndt U (2010). Impact of the routine varicella vaccination programme on varicella epidemiology in Germany. *Euro Surveillance*.

[14] Spackova M, Muehlen M, Siedler A (2010). Complications of varicella after implementation of routine childhood varicella vaccination in Germany. *Pediatric Infectious Disease Journal*.

